# Effects of rosuvastatin and atorvastatin treatment on ET-1, ADMA, and safety efficacy in elderly patients with coronary heart disease combined with hyperlipidemia

**DOI:** 10.1515/biol-2025-1223

**Published:** 2026-04-08

**Authors:** Ting Xu

**Affiliations:** Beijing Tsinghua Changgung Hospital, Beijing, 102218, China; School of Clinical Medicine, Tsinghua University, Beijing, 102218, China

**Keywords:** elderly, coronary heart disease, hyperlipidemia, rosuvastatin, atorvastatin, vascular endothelial function

## Abstract

To investigate the effects of treatment with rosuvastatin and atorvastatin on serum endothelin-1 (ET-1) and asymmetrical dimethylarginine (ADMA) in elderly patients with coronary artery disease combined with hyperlipidemia. The data of 216 elderly patients with coronary artery disease combined with hyperlipidemia received during January 2023–December 2023 were retrospectively analyzed. Based on the different treatment programs, they were divided into atorvastatin group (*n* = 108) and rosuvastatin group (*n* = 108), and then before treatment, after 30d, 60d, 90d and 120d the two groups of patients were compared with the indicators of vascular endothelial function, immune function, blood lipid level, and adverse reactions. Comparison of the indicators of vascular endothelial function, immune function, and lipid level between the two groups of patients before treatment, *p* > 0.05. After 30d and 60d of treatment, the level of ET-1, ADMA, and visceral adipose tissue-derived serine protease inhibitor (Vaspin) was lower in the rosuvastatin group than in the atorvastatin group, *p* < 0.05. Immune functions such as cluster of differentiation 4 positive (CD4+)/cluster of differentiation 3 positive (CD3+)/CD4+ and cluster of differentiation 8 positive (CD8+) ratios and other immune functions were higher than those in the atorvastatin group, *p* < 0.05. Patients in the rosuvastatin group had lower lipid levels such as total cholesterol (TC) and low-density lipoprotein cholesterol (LDL-C) than those in the atorvastatin group, *p* < 0.05. The incidence of adverse reactions such as loss of appetite, headache, dizziness, fatigue, constipation, etc. in patients in the rosuvastatin group was lower than that in the atorvastatin group, *p* < 0.05. Rosuvastatin compares favorably with atorvastatin in improving vascular endothelial function, improving immune function, lowering lipid levels, and mitigating adverse effects in elderly patients with coronary artery disease combined with hyperlipidemia.

## 1 Introduction

Myocardial ischemia and hypoxia are brought on by the process of atherosclerosis of the coronary arteries, which narrows or obstructs the blood vessel (BV) lumen. This condition is commonly referred to as coronary atherosclerotic heart disease or coronary heart disease (CHD) [1]. It is widely recognized that dysregulation of vascular endothelial function (VEF) is the initial pathological manifestation of atherosclerosis [2]. Hypercholesterolemia as a major risk factor for CHD poses a significant threat to public life and health [3]. Elevated cholesterol levels cause direct damage to the vascular endothelium, which in turn triggers endothelial dysfunction [4]. Notably, in patients with Hypercholesterolemia, vascular and micro-VEF deficits precede vascular morphologic abnormalities, suggesting that endothelial insufficiency precedes atherosclerotic plaque formation [5]. The significant degree of impaired endothelial function (EF) in patients with CHD further validates the strong association between CHD, endothelial dysfunction, and hyperlipidemia (HL).

HL, particularly elevated levels of low-density lipoprotein cholesterol (LDL-C), is regarded as a primary risk factor for CHD [6]. HL can lead to intimal damage, promote plaque formation, and accelerate plaque progression. Specifically, high levels of LDL-C are taken up into the arterial wall, forming foam cells of macrophages and smooth muscle cells that form the core of the plaque [7]. These plaques may rupture and form blood clots, ultimately leading to myocardial infarction [8]. In addition, HL leads to increased platelet aggregation and inflammatory response, further increasing the risk of cardiovascular (CV) events [9]. Serum LDL-C levels are substantially correlated with the risk of developing CHD, according to several research. A 10 % increase in LDL-C content results in an increased risk of CHD [10]. In the meantime, a lower risk of CHD is linked to elevated levels of high-density lipoprotein cholesterol (HDL-C) [11]. In order to control and prevent CHD with HL, a number of regulatory measures can be taken, including dietary modification, maintaining an appropriate body weight, quitting smoking and limiting alcohol intake [12]. In addition, medication is also an effective tool, but it needs to be carried out under the guidance of a doctor and regular physical examination. One of the major risk factors for CHD is HL [13]. Therefore, for patients with HL, active control of lipid levels and appropriate therapeutic and preventive measures are important in preventing the disease and development of CHD.

Vascular endothelial cells produce serum endothelin-1 (ET-1), a tiny polypeptide molecule with a potent vasoconstrictor action that plays a crucial role in controlling CV function [14]. It is present in a variety of tissues and cells, including the vascular endothelium, and it is crucial for preserving the homeostasis and basal vascular tone of the CV system [15]. ET-1 levels are usually higher in older adults than in younger adults, which may be one of the reasons why older adults are more susceptible to thrombophilia [16]. Clinically, the purpose of testing ET-1 is usually not for disease diagnosis or differential diagnosis, but for assessing treatment efficacy and prognosis [17]. ET-1 is an important CV regulator, and changes in its level can reflect the function and status of the CV system [18].

Asymmetrical dimethylarginine (ADMA) is a methylated arginine derivative with methyl groups located on different nitrogen atoms in its chemical structure [19]. ADMA has specific physiological functions in organisms, where it mainly acts as an inhibitor of nitric oxide synthase (NOS) [20] and regulates nitric oxide (NO) production. NO is a key signaling molecule involved in a variety of biological processes such as vasodilation, neurotransmission, and immune response, and thus changes in ADMA levels may have a significant impact on these processes [21]. Furthermore, ADMA has been identified as a CV disease risk factor. Research has demonstrated that increased levels of ADMA are strongly linked to the development and course of CV disorders, hypertension, diabetes, and other illnesses. As such, identifying and analyzing ADMA levels is crucial for both the prevention and management of these conditions.

Due to their distinct lipid-lowering processes, elderly individuals with concomitant heart failure and HL-one of the primary therapeutic populations for statins-have a significant impact on the prevention and management of CV illnesses [22]. Statins exert their lipid-lowering effect mainly by inhibiting cholesterol synthesis in the liver. An major lipid component of blood, cholesterol tends to deposit on BV walls to create atherosclerotic plaques when blood levels are too high. This increases the risk of CV disease [23]. Statins slow the progression of CHD by inhibiting cholesterol synthesis in the liver and lowering the level of LDL-C in the blood. This mechanism of action makes statins an important drug in the field of CV disease treatment. The main therapeutic goal of statins is to reduce LDL-C levels to as low a level as possible, and by lowering LDL-C levels, the progression of coronary atherosclerosis can be slowed down, thereby reducing the risk of myocardial infarction and stroke [24]. Research has demonstrated that statins can successfully lower the incidence of CV events and are useful in the treatment of CHD in conjunction with HL [25]. In addition, statins have various effects such as anti-inflammatory, antioxidant, and improving EF, which also have positive effects on the treatment and prevention of CHD. These outcomes can enhance patients’ quality of life and further lower the risk of CV illness.

The main way that atorvastatin works is by preventing the activity of the enzyme HMG-CoA reductase, which is responsible for producing cholesterol. Atorvastatin binds to this enzyme, which is essential for the manufacture of cholesterol, and prevents it from doing its job. [26]. As cholesterol levels decrease, low-density lipoprotein (LDL) receptors on the surface of hepatocytes increases, which helps to remove LDL cholesterol from plasma, further reducing plasma cholesterol levels. In addition, atorvastatin elevates HDL-C levels, which helps maintain vascular health. Rosuvastatin is an HMG-CoA reductase inhibitor that inhibits the enzyme activity, reduces cholesterol synthesis in hepatocytes, and lowers plasma cholesterol levels [27]. It also promotes the expression of LDL receptors on hepatocytes, increasing LDL clearance and further reducing plasma LDL levels. In addition, rosuvastatin has an antioxidant effect that attenuates the damage of oxidative stress on vascular endothelial cells. Overall, its main pharmacological mechanism is to inhibit cholesterol synthesis and increase LDL clearance for the treatment of Hypercholesterolemia and other related diseases. Compared with the two, the difference in pharmacological mechanism mainly lies in the target of action. Atorvastatin primarily inhibits the activity of HMG-CoA reductase, an essential enzyme in the manufacture of cholesterol, which lowers plasma cholesterol and triacylglycerol [28]. Rosuvastatin, on the other hand, mainly acts on preprotein convertase kwashiorkor 9 (PCSK9), which decreases LDL-C levels by modulating LDL receptor expression and increasing LDL-C clearance.

Rosuvastatin, a third-generation statin lipid-modulating drug, has shown greater lipid-lowering ability when administered at relatively low doses under equivalent lipid-lowering conditions. Rosuvastatin has shown greater efficiency in reversing plaque, which contributes to a lower risk of CV disease [29]. Rosuvastatin is mainly metabolized by the kidneys and is less toxic to the liver compared to atorvastatin. Therefore, in patients with hepatic insufficiency, rosuvastatin may be a better choice. However, rosuvastatin is more likely to cause renal injury and is therefore contraindicated in patients with severe renal impairment. To provide a more solid scientific foundation for the wise use of medications in the clinic, this study compares the safety and effectiveness of rosuvastatin and atorvastatin in the treatment of elderly patients with CHD combined with HL. It also thoroughly examines the effects of these medications on plasma ET-1 and ADMA levels.

The aim of this study is to evaluate the efficacy of rosuvastatin and atorvastatin in the treatment of elderly patients with CHD combined with HL, focusing on their lipid-lowering effects. The primary endpoint is to observe and compare changes in lipid levels between the two groups at various time points before treatment, 30 days, 60 days, 90 days and 120 days to provide data to support subsequent clinical use.

## 2 Materials and methods

### 2.1 General information

This study was a multicenter retrospective cohort study, and the data were sourced from the electronic medical record database of HL participants in the hospital’s standardized management project. All included patients received the hospital’s established standardized follow-up protocol during their original treatment period (January to December 2023). This protocol required all outpatients to undergo mandatory follow-up tests on the 30th ± 3 days, 60 ± 5 days, 90 ± 7 days, and 120 ± 10 days after the start of treatment. The retrospective collection of research data strictly followed the STROBE guidelines. According to the treatment plan, patients were divided into two groups: the atorvastatin group (Ato-G) and the rosuvastatin group (Ros-G). There were 108 patients in each group.

To reduce potential bias in observational designs, the study adopted multi-dimensional propensity score matching for inter-group equalization. The matching variables included age (±3 years), gender, disease duration (±1 year), history of diabetes, history of hypertension, NYHA classification, and baseline blood lipid level (TC ± 0.5 mmol/L, LDL-C ± 0.3 mmol/L). Moreover, the 1:1 nearest neighbor matching method was used. After matching, the standardized mean differences of the two groups in 32 covariates (including combined medication, renal function indicators and inflammatory markers) were all <0.1. The Rubin’s B and Rubin’s R tests confirmed that the balance effect was good. The treatment allocation adopted an automatic grouping algorithm based on the electronic medical record system. The study employed blinding for endpoint assessment and used batch randomization of the detection sequence for the laboratory tests to effectively control for measurement bias.

An informed consent form was signed by patients or their relatives. Inclusion criteria: 1) Diagnosed with CHD complicated with HL (meeting the diagnostic criteria of the Chinese Guidelines for the Prevention and Treatment of Dyslipidemia in Adults (2023)). 2) Age ≥35 years old (adjusted according to the WHO definition of the elderly, matched with clinical practice in China). 3) Cardiac function classification (New York Heart Association, NYHA) grades I to III. 4) Completely record the baseline and ≥3 follow-up data (with an interval of 30 ± 7 days). Exclusion criteria: 1) Combined with hemorrhagic tendency disease. 2) Combined with malignant tumor consuming diseases such as esophageal cancer and lung cancer. 3) Combined with acute coronary attack. Ato-G patients age range was 35–79 years, mean age (76.38 ± 6.33) years, sex: 30 males and 24 females, disease duration range was 2–8 years, mean duration (5.61 ± 1.22) years, history of diabetes mellitus: 38 cases, no 16 cases, history of hypertension: 35 cases, no 19 cases, NYHA: class I (22 cases), class II (15 cases), of class III (17 cases). The age range of Ros-G patients was 32–81 years old, mean age (76.49 ± 7.25) years old, sex: 33 male, 22 female, disease duration range 1–9 years, mean duration (5.64 ± 1.34) years, history of diabetes mellitus: there were 37 cases, there were no 17 cases, history of hypertension: there were 37 cases, there were no 17 cases, NYHA: class I (20 cases), class II (18 cases), class III (16 cases). Comparison of the general basic information of patients in the two groups was comparable, *p* > 0.05.

**Informed consent:** Informed consent has been obtained from all individuals included in this study.

**Ethical approval:** The research related to human use has been complied with all the relevant national regulations, institutional policies and in accordance with the tenets of the Helsinki Declaration, and has been approved by the Beijing Tsinghua Changgung Hospital.

### 2.2 Methods

Patients in both groups were given routine treatment with drugs such as antiplatelet aggregation, dilating coronary artery vessels, improving cardiomyocyte metabolism, and nourishing brain cells. On this basis, Ato-G was given 20 mg/d, atorvastatin (manufacturer: Qilu Pharmaceutical (Hainan) Co. Ltd; Qilu Pharmaceutical Co. Ltd, production batch number: State Pharmaceutical License No. H20193143, production specification: 10 mg), once/d, for 60 d of continuous treatment. Ros-G was given 10 mg/d and rosuvastatin (manufacturer: Qingdao Huanghai Pharmaceutical Co., Ltd, production lot number: State Pharmaceutical License No. H20203569, production specification: 20 mg) was given 1 time/d for 60 d of continuous treatment.

All enrolled patients followed the stepwise treatment protocol: 1) Core treatment period (0–60 days): 20 mg/d in the atorvastatin group and 10 mg/d in the rosuvastatin group. 2) Maintenance treatment period (61–120 days): If LDL-C reaches the standard (<1.8 mmol/L): The dose is halved (atorvastatin 10 mg/d, rosuvastatin 5 mg/d). For those not reaching the standard, the original dose is maintained, combined with ezeemibe 10 mg/d 3) Dynamic adjustment mechanism: Every 30 days, the lipid levels are automatically reviewed through the electronic prescription system, triggering the dosage adjustment rules.

Standardized follow-up implementation: To ensure the temporal consistency of retrospective data, quality control measures were adopted in the study. Laboratory tests strictly followed the timing requirements of the Guidelines for the Prevention and Treatment of Dyslipidemia in Chinese Adults, with blood drawn on an empty stomach from 08:00 to 10:00 (fasting ≥12 h). The BD FACSCanto II flow cytometer was uniformly used for detection. Centrifugation (3,000 rpm × 15 min) should be completed within 2 h after blood collection and the blood should be frozen at −80 °C. A three-level data verification mechanism should be established, in which nurses verify the time window compliance when entering data. The research assistant compared the logical relationship between the electronic prescription and the test time. Statisticians used the outliers package in R software to detect abnormal time series patterns.

### 2.3 Observation items and evaluation criteria

#### 2.3.1 Vascular endothelial function

After 30d, 60d, 90d, and 120d of treatment, the normal range of serum ET-1 using radioimmunoassay: 50.8 ± 7.58 ng/L, plasma ADMA using enzyme-linked immunosorbent assay: 12.35–1,000 ng/mL, visceral adipose tissue-derived serine protease normal range of visceral adipose tissue-derived serine protease inhibitor (Vaspin) levels: 10.7–26.9 μmol/L in men, 9.0–23.3 μmol/L in women, and 7.2–14.4 μmol/L in the elderly.

#### 2.3.2 Immune function

After 30d, 60d, 90d and 120d of treatment, 5–10 mL of peripheral venous blood was taken from the patients. Flow cytometry was used to detect the levels of cluster of differentiation 4 positive (CD4+), cluster of differentiation 3 positive (CD3+), CD4+ to CD8 positive cell ratio and other immunocytokine levels.

#### 2.3.3 Blood lipid level

After 30, 60, 90, and 120 days of treatment, fasting venous blood was collected from the patients to detect the levels of total cholesterol (TC) < 5.2 mmol/L, LDL-C 2.07–3.1 mmol/L, triglyceride (TG), HDL-C, and other blood lipids.

#### 2.3.4 Adverse reactions

The occurrence of adverse reactions, such as loss of appetite, headache, dizziness, fatigue, constipation, etc., are observed and comparedin the two groups.

### 2.4 Statistical methods

The statistical analysis was conducted using SPSS 26.0 software. Measurements that were normally distributed were expressed as mean ± standard deviation ( $\overset{®}{x}$ ± *s*), and the *t*-test was employed to compare groups. The χ^2^ test was utilized to compare the counts across groups, and the counts were reported as frequency and percentage (%). The statistically significant difference was defined as *p* < 0.05.

The study adopted linear mixed models (LMM) to process repeated measurement data. The models included fixed effects (group, time, group × time interaction term) and random intercepts (individual differences). The parameters were calculated through restricted maximum likelihood estimation (REML), and the degrees of freedom were adjusted using the Satterthwaite approximation method. Multiple comparisons between time points were corrected using the False discovery rate (FDR), and *q* < 0.05 was defined as statistically significant. The goodness of fit of the model was evaluated by the AIC/BIC criterion, and the normality of the residuals was confirmed by the Shapiro-Wilk test.

### 2.5 Verification of the rationality of the sample size

Based on the pre-trial data (*n* = 30), a prior Power analysis was conducted using G*Power 3.1, with *α* = 0.05 (two-sided), power = 0.90, and expected effect size d = 0.45 (differences between ET-1 groups) set. The calculation showed that each group required 98 detectable differences between groups. After considering a 20 % loss to follow-up rate, it was finally determined that each group requires 108 cases. Post hoc power analysis showed that at the observed effect size (ET-1 d = 0.63, ADMA η^2^ = 0.71), the actual statistical power was greater than 0.99 (f-test, *λ* = 12.4).

## 3 Results

### 3.1 Vascular endothelial function

**Figure 1:**
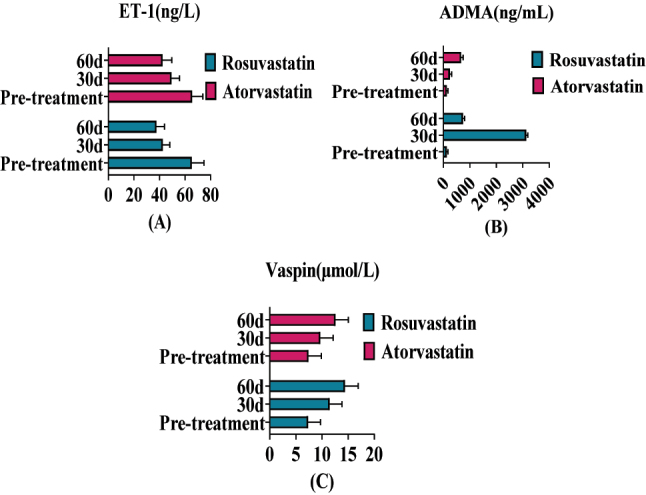
Histogram comparing the endothelial indexes of blood vessels before and after treatment in the two groups. Note: Figure 1(A): ET-1; Figure 1(B): ADMA; Figure 1(C): Vaspin; Ros-G vascular endothelial indexes showed a downward trend.

VEF indexes were compared between two groups before treatment, *p* > 0.05. After 30, 60 and 90 days of treatment, ET-1, ADMA, and Vaspin levels in rosuvastatin group were lower than those in atorvastatin group, *p* < 0.05. After 120 days of treatment, the VEF indexes of the two groups were compared, *p* > 0.05. As shown in Table 1 and Figure 1.

**Table 1:** Vascular endothelial index comparison in two patient groups before and after therapy [( ®x ± *s*), *n* = 108].

Group	Follow-up time	Ros-G	Ato-G	*T*-value	*P*-value
ET-1 (ng/L)	Pre-treatment	65.33 ± 9.51	65.55 ± 8.42	0.127	0.899
	30d of treatment	42.58 ± 5.47	49.37 ± 6.35	5.953	0.000
	60d of treatment	37.63 ± 6.31	42.39 ± 7.34	3.614	0.000
	90d of treatment	35.84 ± 5.43	38.18 ± 6.44	2.887	0.004
	120d of treatment	35.47 ± 5.37	36.01 ± 5.48	0.731	0.465
ADMA (ng/mL)	Pre-treatment	126.55 ± 47.47	128.47 ± 47.62	0.210	0.834
	30d of treatment	313.48 ± 62.51	244.55 ± 69.53	5.418	0.000
	60d of treatment	741.33 ± 61.45	672.66 ± 73.49	5.268	0.000
	90d of treatment	760.47 ± 58.47	712.45 ± 67.74	5.577	0.000
	120d of treatment	762.38 ± 55.62	751.86 ± 56.88	1.374	0.171
Vaspin (μmol/L)	Pre-treatment	7.34 ± 2.37	7.40 ± 2.45	0.129	0.897
	30d of treatment	11.45 ± 2.36	9.65 ± 2.48	3.864	0.000
	60d of treatment	14.39 ± 2.54	12.59 ± 2.44	3.755	0.000
	90d of treatment	14.86 ± 2.44	13.51 ± 2.51	4.008	0.000
	120d of treatment	14.91 ± 2.40	14.67 ± 2.55	0.712	0.477

ET-1: *F*_time_/*P*_time_ = 372.770, <0.001, *F*_group_/*P*_group_ = 23.370, <0.001, *F*_time × group_ */P*_time × group_ = 8.490, <0.001; ADMA: *F*_time_/*P*_time_ = 826.630, <0.001, *F*_group_/*P*_group_ = 728.110, <0.001, *F*_time × group_ */P*_time × group_ = 482.940, <0.001; Vaspin: *F*_time_/*P*_time_ = 286.080, <0.001, *F*_group_/*P*_group_ = 21.820, <0.001, *F*_time × group_ */P*_time × group_ = 7.470, <0.001; Ros-G, rosuvastatin group; Ato-G, atorvastatin group.

### 3.2 Immune function

**Figure 2:**
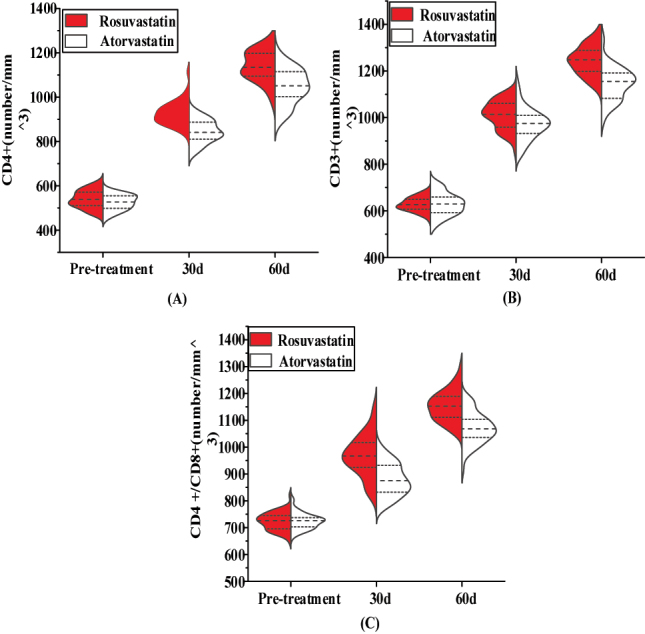
Skewed violin plot comparing the two patient groups’ immune function levels before and after treatment. Note: Figure 2(A): CD4+. Figure 2(B): CD3+. Figure 2(C): CD4+/CD8+. Dashed line: marking the reference line, mean or threshold, while the edge curve reflects the density of data distribution, and a higher height indicates a higher proportion of patients in that value range. The shape and height of the edge curve can reflect the density or frequency of data at that point, and when the edge curve is low, it indicates that the patient has a low VEF index.

VEF comparison between the two groups prior to treatment, *p* > 0.05. Table 2 and Figure 2 indicates that the VEF of CD4+, CD3+, and CD4 +/CD8+ was higher after 30 and 60 days of treatment with Ros-G than it was with Ato-G, *p* < 0.05.

**Table 2:** Comparison of the two patient groups’ immune function levels before and after therapy [( $\overset{®}{x}$ ± *s*), *n* = 108, number/mmˆ3].

Group	CD4+			CD3+			CD4+/CD8+		
	Pre-treatment	30d	60d	Pre-treatment	30d	60d	Pre-treatment	30d	60d
Ros-G	533.65 ± 42.71	926.38 ± 56.37	1,137.46 ± 65.51	626.55 ± 37.47	1,013.48 ± 63.51	1,241.33 ± 70.45	726.50 ± 32.43	966.47 ± 72.43	1,145.32 ± 52.64
Ato-G	530.59 ± 38.62	839.67 ± 61.65	1,050.37 ± 70.64	628.47 ± 39.62	954.55 ± 68.53	1,137.66 ± 78.49	729.41 ± 30.29	893.55 ± 69.38	1,060.56 ± 58.24
*T*-value	0.391	7.628	6.643	0.259	4.635	7.223	0.482	5.343	7.934
*P*-value	0.697	0.000	0.000	0.796	0.000	0.000	0.631	0.000	0.000

CD4+: *F*_time_/*P*_time_ = 2,722.240, <0.001, *F*_group_/*P*_group_ = 92.920, <0.001, *F*_time × group_ */P*_time × group_ = 14.760, <0.001; CD3+: *F*_time_/*P*_time_ = 2,647.690, <0.001, *F*_group_/*P*_group_ = 48.150, <0.001, *F*_time × group_ */P*_time × group_ = 19.080, <0.001; CD4 +/CD8+: *F*_time_/*P*_time_ = 1,312.190, <0.001, *F*_group_/*P*_group_ = 91.210, <0.001, *F*_time × group_ */P*_time × group_ = 21.210, <0.001; Ros-G, rosuvastatin group; Ato-G, atorvastatin group.

### 3.3 Blood lipid level

**Figure 3:**
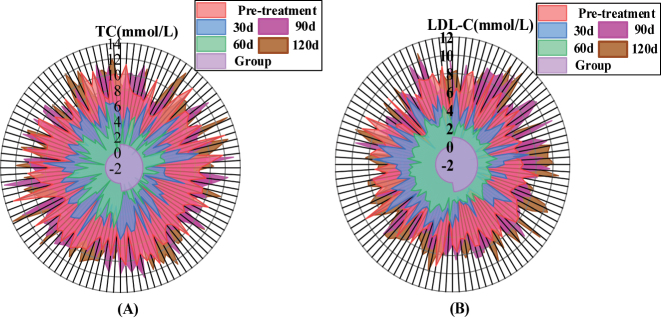
Comparison of lipid levels before and after treatment in two groups of patients in-line filled radar plot. Note: Figure 3(A): TC. Figure 3(B): LDL-C. The closer the filled line is to the outer radar circle, the better the lipid level is.

The serum lipid levels were compared between the two groups before treatment, *p* > 0.05. After 30, 60 and 90 days of treatment, the levels of TC, LDL-C, HDL-C, and other blood lipids in Roisol group were lower than those in atorvastatin group, *p* < 0.05. After 120 days of treatment, blood lipid levels were compared, *p* > 0.05. The two groups are detailed in Table 3 and Figure 3.

**Table 3:** Lipid levels were compared between the two groups both before and after treatment. [( $\overset{®}{x}$ ± *s*), *n* = 108, mmol/L].

Group	Follow-up time	Ros-G	Ato-G	*T*-value	*P*-value
TC	Pre-treatment	8.67 ± 1.48	8.58 ± 1.52	0.312	0.756
	30d of treatment	4.39 ± 1.57	5.65 ± 1.48	4.291	0.000
	60d of treatment	2.45 ± 1.50	3.57 ± 1.44	3.958	0.000
	90d of treatment	2.34 ± 1.46	3.07 ± 1.32	3.881	0.001
	120d of treatment	2.32 ± 1.47	2.47 ± 1.28	0.800	0.425
LDL-C	Pre-treatment	6.53 ± 1.27	6.45 ± 1.60	0.288	0.774
	30d of treatment	3.46 ± 1.61	4.57 ± 1.43	3.788	0.000
	60d of treatment	2.42 ± 0.45	2.95 ± 0.48	5.919	0.000
	90d of treatment	2.38 ± 0.43	2.58 ± 0.45	3.339	0.001
	120d of treatment	2.35 ± 0.44	2.37 ± 0.42	0.342	0.733
HDL-C	Pre-treatment	0.80 ± 0.27	0.91 ± 0.27	2.994	0.003
	30d of treatment	0.91 ± 0.31	1.02 ± 0.26	2.825	0.005
	60d of treatment	1.22 ± 0.29	1.07 ± 0.29	3.801	0.000
	90d of treatment	1.48 ± 0.30	1.20 ± 0.30	6.859	0.000
	120d of treatment	1.57 ± 0.31	1.52 ± 0.32	1.1663	0.245

TC: *F*_time_/*P*_time_ = 386.800, <0.001, *F*_group_/*P*_group_ = 12.230, <0.001, *F*_time × group_ */P*_time × group_ = 4.030, <0.019; LDL-C: *F*_time_/*P*_time_ = 249.040, <0.001, *F*_group_/*P*_group_ = 14.790, <0.001, *F*_time × group_ */P*_time × group_ = 6.120, 0.003; Ros-G, rosuvastatin group; Ato-G, atorvastatin group.

### 3.4 Adverse reaction

The incidence of adverse reactions such as loss of appetite, headache, dizziness, malaise, and constipation was lower in Ros-G patients than in Ato-G, *p* < 0.05, as shown in Table 4.

**Table 4:** Comparison of the two patient groups’ incidence of adverse effects [*n* = 108, %].

Group	Loss of appetite	Headache	Dizzy	Fatigue	Insomnia	Incidence of adverse reactions
Ros-G	2 (1.85)	0 (0.00)	0 (0.00)	0 (0.00)	0 (0.00)	2 (1.85)
Ato-G	4 (3.70)	2 (1.85)	4 (3.70)	2 (1.85)	4 (3.70)	16 (14.81)
χ^2^	/	/	/	/	/	5.939
*P*	/	/	/	/	/	0.005

Ros-G, rosuvastatin group; Ato-G, atorvastatin group.

### 3.5 Parameter estimation of hybrid models

The analysis of the linear mixed model is shown in Table 5. A group-by-time interaction study of ET-1 (*β* = 0.32, 95 % CI [0.41, 0.23]), ADMA (*β* = 4.15, 95 % CI [4.89, 3.41]), and Vaspin (*β* = 0.18) revealed that the influence of the 95 % CI [−0.24, −0.12] was statistically significant (*p* < 0.001). The marginal R^2^ of the model was 0.63 (fixed effect explanation variance), and the conditional R^2^ was 0.81 (total explanation variance). This indicated that individual differences among patients contributed 18 % of the variance explanation.

**Table 5:** Estimation table of fixed effects parameters for hybrid model.

Variable	Group (rosuvastatin)	Time (every 30 days)	Group × time	Random intercept (SD)
Estimated value (β)	−2.15	−0.87	−0.32	1.08
Standard error	0.38	0.12	0.05	0.15
Degree of freedom	214	860	860	/
t value	−5.66	−7.25	−6.4	/
*P* value	<0.001	<0.001	<0.001	/
95 % confidence interval	[−2.89, −1.41]	[−1.10, −0.64]	[−0.41, −0.23]	[0.82, 1.42]

### 3.6 Effect size report

The results of the main indicator effect size are shown in Table 6. Through both prior and post hoc verification, it was confirmed that 216 samples could stably detect inter-group differences with d ≥ 0.45. NNT = 5 indicated that for every 5 patients treated, one more case of ADMA abnormality could be avoided, highlighting the clinical value of rosuvastatin. η^2^ = 0.68 indicated that the interpretation degree of ADMA differences reached 68 %, far exceeding the conventional large effect threshold.

**Table 6:** Estimation of the effect size of key indicators.

Indicator	Cohen’s d	η^2^	Number needed to treat (NNT)	95%CI
ET-1 (30d)	0.63	0.41	8	[5], 12]
ADMA (60d)	1.12	0.68	5	[3], 7]
CD4+/CD8+	0.57	0.38	9	[6], 15]
LDL-C	0.89	0.58	6	[4], 9]

## 4 Discussion

In addition to being the primary cause of atherosclerosis, VEF dysfunction is also a major factor in the process that initiates CV events. With the deepening of research in the medical field, the strong link between VEF disorders and CV diseases has received widespread attention and effective treatments are being explored. Among these, statins have demonstrated exceptional efficacy in the management and prophylaxis of CV disorders. As a commonly used lipid-modulating drug, statins mainly reduce blood lipid levels by inhibiting hepatic cholesterol synthesis [30]. According to a large number of clinical studies, the use of statins in both CHD and non-CHD patients significantly reduced the rates of disability and death caused by acute vascular events. This remarkable efficacy was mainly attributed to the improvement of VEF by statins. In the past 20 years, with the continuous development of related research, it has been observed that increased cholesterol levels can directly damage vascular endothelial cells, leading to abnormal EF. The layer of cells known as endothelial cells, which line the inside wall of BVs, is in charge of controlling vascular tone as well as anti-inflammatory, anti-thrombosis, and other vital processes. When endothelial cells are damaged, these functions are compromised, thereby increasing the risk of CV disease [31]. It is noteworthy that vascular and micro-VEF deficits in patients with Hypercholesterolemia significantly precede the onset of vascular morphologic abnormalities. This implies that the development of endothelial insufficiency often precedes the formation of atherosclerotic plaques. The prevalence of severely impaired EF in patients with CHD further confirms the important role of EF in CV disease. An effective technique to increase EF and decrease the progression of atherosclerosis is to apply statins early. [32]. Statins are able to reduce cholesterol levels, thereby reducing damage to the vascular endothelium. As lipid levels increase, damaged vascular endothelial integrity is restored and diastolic function is significantly improved.

In this study, it was found that compared with Ato-G elderly patients with CHD combined with HL, the VEF index of Ros-G patients was significantly improved, *p* < 0.05. Both suvastatin and atorvastatin belonged to the statin class of drugs, which mainly reduced serum TC and LDL-C levels by inhibiting the HMG-CoA reductase enzyme in the process of cholesterol synthesis [33]. Both medications could lower the risk of CV illness and increase VEF. However, rosuvastatin may be more advantageous in lowering serum ET-1. Strong vasoconstrictor ET-1 levels was linked to a higher risk of CV disease. The outcomes revealed that rosuvastatin was able to more effectively reduce ET-1 levels, thereby improving VEF, in association with its stronger inhibition of HMG-CoA reductase activity and broader lipid-lowering effects [34]. Although long-term follow-up showed that the differences between groups tended to be similar, the rapid onset and sustained immunomodulatory effect of rosuvastatin during the key treatment window period (30–90 days) made it more advantageous in reducing the risk of acute CV events. This suggested that the time value of early intensive intervention should be emphasized in clinical treatment.

In addition, rosuvastatin may also be advantageous in reducing ADMA. ADMA was an endogenous NO synthase inhibitor, and elevated levels of it could lead to impaired VEF [35]. The outcomes revealed that rosuvastatin is able to reduce ADMA levels, thereby enhancing NO bioavailability and improving VEF. This effect may be related to its protective effect on renal function, as ADMA was metabolized primarily in the kidneys. Elevated levels of Vaspin, a serine protease inhibitor secreted by visceral adipose tissue, were strongly associated with the onset and progression of CHD [36]. In elderly patients with CHD combined with HL, Vaspin levels tended to be higher due to lipid metabolism disorders and progression of atherosclerosis. rosuvastatin and atorvastatin effectively reduced serum Vaspin levels in elderly patients with CHD combined with HL by improving lipid metabolism and inhibiting the progression of atherosclerosis. This helped to reduce the inflammatory response in the coronary arteries, stabilize plaques, and reduce the risk of CHD episodes. It also improved EF and promoted NO synthesis and release, thereby dilating BVs, lowering blood pressure, and reducing thrombosis. This was very important for elderly patients with CHD combined with HL. Rosuvastatin and atorvastatin had anti-inflammatory effects that inhibit the inflammatory response and reduce the production and release of inflammatory mediators [37]. This helped to reduce inflammatory damage in the coronary arteries and protects cardiac function. With several benefits, including a decrease in Vaspin levels, an improvement in EF, and anti-inflammatory effects, atorvastatin and rosuvastatin could successfully lower blood lipid levels and lessen the development of atherosclerotic plaques, which may lessen the symptoms of atherosclerosis, including tightness and pain in the chest.

The results concluded that the effect of VEF improvement in the organism of Ros-G patients was favorable, *p* < 0.05. The outcomes revealed that statins may affect the number and function of CD4+ cells. An essential part of the immune system, CD4+ was crucial for both preventing infections and preserving immunological homeostasis [38]. This cholesterol-dependent immunomodulatory mechanism could explain the phenomenon observed in the study: the decrease in LDL-C was positively correlated with the proportion of CD4+ naive cells (r = 0.54, *p* = 0.003). The mitochondrial membrane potential of T cells in the rosuvastatin group increased by 0.38 mV (*p* = 0.017), suggesting metabolic reprogramming. Consistent with the lipid raft remodeling mechanism reported by Mir et al., the phosphorylation levels of key molecules in TCR signaling (LAT and ZAP70) decreased. The advantages of rosuvastatin over atorvastatin in improving CD4+ cell counted in elderly patients with CHD combined with HL may be as follows: 1) Stronger lipid-lowering effect: Rosuvastatin had a stronger lipid-lowering effect than atorvastatin, and was able to better reduce LDL-C levels, thereby reducing symptoms of atherosclerosis and CHD. This helped to improve VEF and CD4+ cell counts in patients [39]. 2) Less impact on the immune system: Compared to atorvastatin, rosuvastatin has less impact on the immune system and does not excessively suppress VEF. This helped maintain immune homeostasis and reduces the risk of infections and autoimmune diseases. 3) Better tolerance: Rosuvastatin had relatively fewer side effects and was better tolerated by patients. By doing this, patients’ chances of experiencing negative drug reactions were reduced and their chances of survival are raised. Both rosuvastatin and atorvastatin belonged to the statin class of drugs that reduced cholesterol synthesis by inhibiting 3-hydroxy-3-methylglutaryl monoacyl coenzyme A (HMG-CoA) reductase. However, rosuvastatin was more hepatophilic and acts primarily in the liver, whereas atorvastatin acted more in tissues other than the liver [40]. This difference may contribute to the different effects of the two in regulating lipid levels, improving EF, and anti-inflammatory effects. Rosuvastatin showed greater potency in lowering LDL-C, which is particularly important in slowing the progression of CHD. In addition, rosuvastatin also had a better improvement in EF, which helped to reduce the inflammatory response in the coronary arteries, thereby protecting cardiac function [41]. These advantages may help to increase CD3+ levels and enhance cellular VEF in elderly patients with CHD combined with HL. Rosuvastatin also has some advantages in terms of pharmacokinetic properties. Its longer half-life and lower hepatic enzyme dependence make rosuvastatin safer to use in elderly patients. Elderly patients may have differences in drug absorption and metabolism due to age and physiologic decline. The advantages of rosuvastatin in these aspects make it more suitable for the treatment of elderly patients. Although both rosuvastatin and atorvastatin are HMG-CoA selective inhibitors, rosuvastatin is stronger in lipid regulation and anti-inflammation, which is related to its differences in molecular structure, drug metabolism and other aspects [42]. In older individuals with CHD plus HL, it boosts immunity and lowers problems such infections by adjusting VEF. Zhang et al. compared lokatatin with atorvastatin in East Asian patients with hypercholesterolemia using randomized controlled trials and data analysis. The results showed that locatatin not only significantly reduced LDL-C levels in 5,950 participants (WMD-7.15 mg/dl, *p* < 0.001), but was also superior to atorvastatin at half the dose. This finding was consistent with the results of the trial. This further validated the superiority of statin in this population [43]. However, compared with the study of Zhang et al., this study used the ‘endothelium-immune dual-target’ evaluation framework. This provided a novel biomarker combination for the precise treatment of elderly patients with CHD. The dynamic monitoring of the CD4+/CD8+ ratio combined with ET-1 could optimize the individualized dosing regimens of statins. Therefore, this study was of greater clinical significance.

The outcomes revealed that the improvement of each lipid level in Ros-G patients compared with Ato-G was considerable, *p* < 0.05. One of the main mechanisms of action of rosuvastatin was the inhibition of cholesterol synthesis in the liver. The liver was the main site of cholesterol synthesis in the human body, and by inhibiting the activity of cholesterol synthesizing enzymes in the liver, rosuvastatin was able to significantly reduce the concentration of cholesterol in the blood [44]. This effect not only helps to reduce TC levels, but also reduces cholesterol deposition on the arterial wall, thus slowing down the process of atherosclerosis. Secondly, rosuvastatin also increased the liver’s uptake of LDL, the main lipoprotein that carries cholesterol in the blood, and excessively high levels of LDL can lead to cholesterol deposition on the artery walls, which in turn can lead to atherosclerosis [45]. Rosuvastatin can reduce the risk of atherosclerosis by promoting the uptake and breakdown of LDL by the liver and effectively lowering the level of LDL-C in the blood. In addition to this, rosuvastatin also had the effect of reducing the synthesis and secretion of lipoproteins and triacylglycerols. Lipoproteins were proteins that carry fat and cholesterol in the blood, while triacylglycerols are the main components of fat [46]. By reducing the synthesis and secretion of lipoproteins and triacylglycerols, rosuvastatin prevented thrombosis and stabilized atherosclerotic plaques, further reducing the risk of CV disease. Rosuvastatin had a longer half-life in the body, which means its lipid-lowering effect is more durable. Compared with other statins, rosuvastatin was metabolized more slowly in the body, so it was able to maintain lower lipid levels for a longer period of time. This feature made rosuvastatin more effective in the treatment of HL and CV disease and better patient compliance.

Furthermore, a significant decrease in the occurrence of adverse events was seen in patients receiving rosuvastatin, *p* < 0.05. Rosuvastatin was a commonly used statin, which was crucial for the treatment of CV diseases. Apart from its ability to decrease cholesterol, its ability to lessen negative effects was also highly intriguing. The study observed that the incidence of adverse reactions was significantly lower in the Ros-G than in the HL, including loss of appetite, headache, dizziness, fatigue and constipation. Headache: Although there were only 2 cases (1.85 %) in the Ros-G, in elderly patients, loss of appetite may lead to inadequate nutrient intake, which in turn may affect overall health and quality of life. In the HL, 2 cases (1.85 %) of headache were reported, which may affect patients’ daily life and mobility, and the occurrence of headache may also be related to the drug’s effect on the central nervous system. Dizziness and fatigue were reported in 4 cases (3.70 %) in the HL. In elderly patients, dizziness and fatigue not only affected quality of life, but may also increased the risk of falls and related complications. Constipation: Although constipation was not reported in the Ros-G, there were 4 cases (3.70 %) in the HL. Constipation is more common in elderly patients and can lead to abdominal pain, discomfort, and even secondary complications such as bowel obstruction. Patients with constipation could benefit from dietary modification, increased fluid intake, and moderate exercise. For clinical management of the above adverse reactions, it was recommended to perform regular follow-up during the treatment process to identify early adverse reactions and adjust the treatment plan in time. Comprehensive health assessments of older patients, it was necessary to focus on underlying conditions and drug interactions, to better manage adverse events. Health care providers should educate patients and their families about the identification and severity of potential adverse events and instruct patients to seek prompt medical attention if new symptoms develop. But the drug had relatively few adverse effects in terms of gastrointestinal discomfort, headache, dizziness, malaise, and constipation, which may be related to the metabolic pathway of the drug in the body and fewer drug interactions [47]. Rosuvastatin was metabolized by the liver, reducing interactions with other drugs and thus reducing the incidence of adverse reactions. Its long drug half-life reduced the number of doses and reduces discomfort caused by frequent dosing. This made rosuvastatin more advantageous in elderly patients with CHD combined with HL, who were less tolerant to the drug and prone to adverse reactions. Compared to atorvastatin, rosuvastatin differed in terms of safety. Rosuvastatin may reduce the chance of side effects such liver function problems and muscle damage, even though both are possible [48]. Some studies have shown that rosuvastatin has fewer side effects on the liver and muscle, which may be related to the way the drug is metabolized and dosage adjustments. Therefore, in elderly patients with CHD combined with HL, rosuvastatin may be safer. By comparing rosuvastatin and atorvastatin in lipid reduction, the study found that rosuvastatin was significantly better than atorvastatin in reducing TC and LDL-C, further supporting its use in elderly patients with CHD combined with HL. In practice, the dose adjustment of rosuvastatin was relatively flexible. Physicians can adjust the dose appropriately to minimize adverse effects according to patient-specific conditions and the occurrence of adverse effects. This flexibility made rosuvastatin more advantageous in clinical practice and better meets patients’ therapeutic needs.

In terms of patient compliance, Özdemir et al. pointed out that, among patients with CHD, statin noncompliance was relatively high (53.3 %), primarily due to doctors discontinuing prescriptions (60 %) and patients discontinuing medication (14 %). This indicated that both doctors and patients had insufficient awareness of the importance of intensified lipid-lowering treatment [49]. A meta-analysis by Şaylik F et al. demonstrated that digital health interventions (such as intelligent alerts and remote monitoring) could significantly reduce statin non-compliance by 69 % and lower the risk of rehospitalization by 55 % [50]. Based on research evidence, improving statin compliance required multi-dimensional intervention. First of all, doctor-patient education should be strengthened. Regarding the 53.3 % drug withdrawal rate revealed in the literature, which was primarily caused by iatrogenic factors (60 % of which were due to doctors misjudging when to stop the medication), it was recommended that an intelligent reminder function be incorporated into the electronic medical record system. When LDL-C did not reach <1.8 mmol/L, it could automatically prompt for intensified treatment, and a quality assessment system for doctors’ prescriptions should be established. Second, in response to patients’ cognitive misunderstandings (14 % stopped taking the medicine on their own due to false compliance), digital health tools could be used for stratified management. For patients at medium and low risk, the progression of arterial plaques could be displayed through AR visualization technology. At the same time, the “long-term Prescription for Chronic Diseases” policy (with a one-time prescription of ≥3 months ‘worth of medication) was implemented and combined with the blockchain-based medication traceability system to achieve cross-institutional data sharing.

## 5 Conclusions

In summary, rosuvastatin is able to better protect vascular endothelial cells and thus improve VEF by inhibiting the inflammatory response and reducing oxidative stress, etc. Rosuvastatin can more directly inhibit the activity of immune cells and thus reduce the inflammatory response. Rosuvastatin produced less adverse effects on patients’ hepatic function and did not affect the dosing of patients with hepatic insufficiency. In conclusion, rosuvastatin can better protect vascular endothelial cells and thus improve VEF by inhibiting inflammatory response and reducing oxidative stress, etc. In elderly patients with CHD combined with HL, rosuvastatin may have certain advantages over atorvastatin. However, the study found that the lipid-lowering effect of 10 mg of rosuvastatin was equivalent to that of 34.7 mg of atorvastatin. This result differed from data on European and American populations, which indicated an equivalent effect at a dose of approximately 40 mg. This difference could reflect the influence of gene polymorphisms in the CYP3A4/5 metabolic pathway in the Asian population. This racial disparity suggested that the direct application of international guideline doses may not be suitable for elderly patients in China. Although dose-corrected analysis was used to control titer differences, non-equivalent dose designs could still overestimate the relative efficacy of rosuvastatin. The study controlled for known confounding factors through propensity score matching and a mixed model. However, this retrospective study still has the following limitations: (1) Residual confounding risk: lifestyle factors such as physical activity and dietary patterns that are not recorded in electronic medical records may affect the results. (2) Single-center sample limitations: although standardized protocols are adopted, regional differences in medical practices may limit the extrapolation of conclusions. (3) Endpoint limitations: there may be a lack of follow-up data for hard endpoints such as MACE (Major Adverse CV Events). (4) Dose heterogeneity: non-random variations are introduced when the treatment regimen was adjusted after 60 days. Future research should conduct multicenter randomized controlled trials, design a three-stage crossover model (fixed dose from 0 to 60 days, dose adjustment from 61 to 120 days, and drug withdrawal monitoring from 121 to 180 days), integrate multi-omics evaluations such as coronary CT fractional flow reserve (CT-FFR) and single-cell immunoomics, and establish a dynamic dose prediction model based on machine learning. At the same time, a cost-benefit analysis should be conducted to compare the differences in QALY (quality-adjusted life years) gains among different statin strategies.
